# Analysis of the equivalence relationship between $l_{0}$-minimization and $l_{p}$-minimization

**DOI:** 10.1186/s13660-017-1590-x

**Published:** 2017-12-21

**Authors:** Changlong Wang, Jigen Peng

**Affiliations:** School of Mathematics and Statistics, Xi’an Jiaotong University, Xi’an, Shaanxi 710049 China

**Keywords:** 60E05, 94A12, 94A20, sparse recovery, null space constant, null space property, $l_{0}$-minimization, $l_{p}$-minimization

## Abstract

In signal processing theory, $l_{0}$-minimization is an important mathematical model. Unfortunately, $l_{0}$-minimization is actually NP-hard. The most widely studied approach to this NP-hard problem is based on solving $l_{p}$-minimization ($0< p\leq1$). In this paper, we present an analytic expression of $p^{\ast}(A,b)$, which is formulated by the dimension of the matrix $A\in\mathbb{R}^{m\times n}$, the eigenvalue of the matrix $A^{T}A$, and the vector $b\in\mathbb{R}^{m}$, such that every *k*-sparse vector $x\in\mathbb{R}^{n}$ can be exactly recovered via $l_{p}$-minimization whenever $0< p< p^{\ast}(A,b)$, that is, $l_{p}$-minimization is equivalent to $l_{0}$-minimization whenever $0< p< p^{\ast}(A,b)$. The superiority of our results is that the analytic expression and each its part can be easily calculated. Finally, we give two examples to confirm the validity of our conclusions.

## Introduction

In sparse information theory, a central goal is to get the sparsest solutions of underdetermined linear systems including visual coding [1], matrix completion [2], source localization [3], and face recognition [4]. All these problems are popularly modeled by the following $l_{0}$-minimization:

1 $$ \min _{x \in\mathbb{R}^{n}} \|x\|_{0} \quad\mbox{s.t.}\quad Ax=b, $$

where $A \in\mathbb{R}^{m \times n}$ is an underdetermined matrix (*i.e*. $m< n$), and $\|x\|_{0}$ is the number of nonzero elements of *x*, which is commonly called $l_{0}$-norm although it is not a true vector norm. If $x\in\mathbb{R}^{n}$ is a unique solution of $l_{0}$-minimization, we also say that *x* can be recovered by $l_{0}$-minimization; we adopt these two statements in this paper.

Since *A* has more columns than rows, the underdetermined linear system $Ax=b$ admits an infinite number of solutions. To find the sparsest one, much excellent theoretical work (see, *e.g*. [5, 6], and [7]) has been devoted to the $l_{0}$-minimization. However, Natarajan [8] proved that $l_{0}$-minimization is NP-hard. Furthermore, it is combinationally and computationally intractable to solve $l_{0}$-minimization directly because of its discrete and discontinuous nature. Therefore, a lot of work put forward some alternative strategies to get the sparsest solution (see, *e.g*. [5, 9–14], and [15]). Among these methods, the most popular one is $l_{p}$-minimization with $0< p\leq1$ introduced by Gribonval and Nielsen [16],

2 $$ \min _{x \in\mathbb{R}^{n}} \|x\|_{p}^{p} \quad\mbox{s.t.}\quad Ax=b, $$

where $\|x\|_{p}^{p}=\sum_{i=1}^{n} |x_{i}|^{p}$. In the literature, $\|x\|_{p}$ is still called the *p*-norm of *x* though it is only a quasi-norm when $0< p<1$ (because in this case it violates the triangle inequality). Due to the fact that $\|x\|_{0}=\lim _{p \to0} \|x\|_{p}^{p}$, $l_{0}$-minimization and $l_{p}$-minimization are collectively called $l_{p}$-minimization with $0\leq p\leq1$ in this paper.

However, to get the sparsest solution of $Ax=b$ via $l_{p}$-minimization, we need certain conditions on *A* and/or *b*, for example, the novel restricted isometry property (RIP) of *A*. A matrix *A* is said to have restricted isometry property of order *k* with restricted isometry constant $\delta_{k} \in(0,1)$ if $\delta_{k}$ is the smallest constant such that

3 $$ (1-\delta_{k})\|x\|_{2}^{2} \leq \|Ax \|_{2}^{2} \leq(1+\delta _{k})\|x \|_{2}^{2} $$

for all *k*-sparse vectors *x*, where a vector *x* is said to be *k*-sparse if $\|x\|_{0}\leq k$.

There exist a lot of sufficient conditions for the exact recovery by $l_{1}$-minimization, such as $\delta_{3k}+3\delta_{4k}<2$ in [10], $\delta_{2k}<{\sqrt{2}-1}$ in [9], and $\delta _{2k}<2(3-\sqrt{2})/7$ in [11]. Cai and Zhang [17] showed that for any given $t\geq\frac{4}{3}$, the condition $\delta _{tk}<\sqrt{\frac{t-1}{t}}$ guarantees recovery of every *k*-sparse vector by $l_{1}$-minimization. From the definition of *p*-norm it seems to be more natural to consider $l_{p}$-minimization with $0< p<1$ instead of $l_{0}$-minimization. Foucart [11] showed that the condition $\delta_{2k}<0.4531$ can guarantee exact *k*-sparse recovery via $l_{p}$-minimization for any $0< p<1$. Chartrand [18] proved that if $\delta_{2k+1} < 1$, then we can recover a *k*-sparse vector by $l_{p}$-minimization for some $p > 0$ small enough. However, it should be pointed out that the problem of calculating $\delta_{2k}$ for a given matrix *A* is still NP-hard.

Recently, Peng, Yue, and Li [7] have proved that there exists a constant $p(A,b)>0$ such that every solution of $l_{p}$-minimization is also a solution of $l_{0}$-minimization whenever $0< p< p(A,b)$. This result builds a bridge between $l_{p}$-minimization and $l_{0}$-minimization, and it is important that this conclusion is not limited by the structure of a matrix *A*. However, the paper [7] does not give an analytic expression of $p(A,b)$. The model of choice of $l_{p}$-minimization is still difficult.

As already mentioned, it is NP-hard to calculate $\delta_{2k}$ for a given matrix $A\in\mathbb{R}^{m\times n}$ and also to calculate these *p*. On the other hand, the possibility of recovery of every *k*-sparse vector by $l_{0}$-minimization is just a necessary condition for the existence of such $\delta_{2k}$, and therefore the results based on $\delta_{2k}$ lead to limitations of practical application.

We have to emphasize that although $l_{p}$-minimization is also difficult due to its nonconvexity and nonsmoothness, a lot of algorithms have been designed to solve $l_{p}$-minimization; see *e.g*. [11, 19], and [20]. Moreover, a reasonable range of *p* in these algorithms is very important. In this paper, we devote ourselves to giving a complete answer to this problem.

Our paper is organized as follows. In Section 2, we present some preliminaries of the $l_{p}$-null space property, which plays a core role in the proof of our main theorem. In Section 3, we focus ourselves on proving the main results of this paper: we present an analytic expression of $p^{\ast}(A,b)$ such that every *k*-sparse vector $x\in \mathbb{R}^{n}$ can be exactly recovered via $l_{p}$-minimization with $0< p< p^{\ast}(A,b)$ as long as *x* can be recovered via $l_{0}$-minimization. Finally, we summarize our findings in the last section.

For convenience, for $x \in\mathbb{R}^{n}$, its support is defined by $\operatorname{support} (x)=\{i:x_{i} \neq0\}$, and the cardinality of a set Ω is denoted by $|\Omega|$. Let $\operatorname{Ker}(A)=\{x \in\mathbb{R}^{n}:Ax=0\}$ be the null space of a matrix *A*, and denote by $\lambda_{\min^{+}}(A)$ the minimal nonzero absolute-value eigenvalue of *A* and by $\lambda_{\max}(A)$ the maximal one. We denote by $x_{\Omega}$ the vector that is equal to *x* on the index set Ω and zero elsewhere and by $A_{\Omega}$ the submatrix the columns of which are the columns of *A* that are in the set index Ω. Let $\Omega^{c}$ be the complement of Ω.

## Preliminaries

To investigate conditions under which both $l_{0}$-minimization and $l_{p}$-minimization have the same unique solution, it is convenient for us to work with a sufficient and necessary condition of the solutions of $l_{0}$-minimization and $l_{p}$-minimization. Therefore, in this preliminary section, we focus on introducing such an condition, namely the $l_{p}$-null space property.

### Definition 1

([16])

A matrix $A \in\mathbb{R}^{m \times n}$ with $m \leq n$ is said to satisfy the $l_{p}$-null space property of order *k* if

4 $$ \|x_{\Omega}\|_{p} < \|x_{\Omega^{c}}\|_{p} $$

for every $x \in \operatorname{Ker}(A)\setminus\{\mathbf{0}\}$ and every set $\Omega \subset\{1,2 ,\ldots, n\}$ with $|\Omega| \leq k$.

In the literature, the null space property usually means the $l_{1}$-null space property. We now indicate the relation between the $l_{p}$-null space property and exact recovery via $l_{p}$-minimization with $0\leq p\leq1$.

### Theorem 1

([16, 21])

*Given a matrix* $A \in\mathbb{R}^{m \times n}$ *with* $m \leq n$, *every* *k*-*sparse vector* $x\in\mathbb{R}^{n}$ *can be recovered via* $l_{p}$-*minimization with* $0\leq p \leq1$ *if and only if* *A* *satisfies the* $l_{p}$-*null space property of order* *k*.

Theorem 1 provides a sufficient and necessary condition to judge whether a vector can be recovered by $l_{p}$-minimization with $0\leq p\leq1$, which is the most important advantage of the $l_{p}$-null space property. However, the $l_{p}$-null space property is difficult to be checked for a given matrix. To reach our goal, we recall the concept of the null space constant (NSC), which is closely related to the $l_{p}$-null space property and offers tremendous help in illustrating the performance of $l_{0}$-minimization and $l_{p}$-minimization.

### Definition 2

([22])

For any $0 \leq p \leq1$ and $k>0$, the null space constant (NSC) $h(p,A,k)$ is the smallest number such that:

5 $$ \sum_{i \in\Omega}|x_{i}|^{p} \leq h(p,A,k) \sum_{i \notin\Omega}|x|^{p} $$

for every index set $\Omega\subset\{1,2,\ldots,n\}$ with $|\Omega| \leq k$ and every $x \in \operatorname{Ker}(A)\setminus\{\mathbf{0}\}$.

Similarly to the $l_{p}$-null space property, NSC also can be used for characterizing the performance of $l_{p}$-minimization. Combining the definition of NSC and the results in [23] and [22], we can derive the following corollaries.

### Corollary 1

*For any* $p \in[0,1]$, $h(p,A,k)<1$ *is a sufficient and necessary condition for recovery of all* *k*-*sparse vectors via* $l_{p}$-*minimization with* $0\leq p\leq1$.

### Proof

The proof is very easy, and we leave it to the readers. □

### Corollary 2

*Given a matrix* $A\in\mathbb{R}^{m \times n}$, *if* $h(0,A,k)<1$, *we have*:

- $\|x\|_{0} \geq2k+1$ *for every* $x \in \operatorname{Ker}(A)\setminus\{\mathbf{0}\}$;
- $k \leq \lceil\frac{n-2.5}{2} \rceil+1$, *where* $\lceil a \rceil$ *represents the integer part of* *a*.

### Proof

(a) We assume that there exists a vector $x\in \operatorname{Ker}(A)\setminus\{\mathbf {0}\}$ with $\|x\|_{0}\leq2k$.

Let $\Omega=\operatorname{support} (x)$. If $\|x\|_{0}\leq k$, then we get that $\| x_{\Omega}\|_{0}\geq\|x_{\Omega^{c}}\|_{0}=0$.

If $k<\|x\|_{0}\leq2k$, we consider an arbitrary set $\widetilde{\Omega }\subset\Omega$ with $|\widetilde{\Omega}|=k$. Then we get that $\| x_{\widetilde{\Omega}}\|_{0}=k\geq\|x_{\widetilde{\Omega}^{c}}\|_{0}$.

According to the definition of $h(p,A,k)$, these two conclusions contradict $h(0,A,k)<1$, and therefore we have that $\|x\|_{0} \geq2k+1$ for any $x \in \operatorname{Ker}(A)\setminus\{\mathbf{0}\}$.

(b) As has been proved in (a), we get that

6 $$ 2k+1\leq\|x\|_{0}\leq n. $$

Due to the integer values of $\|x\|_{0}$ and *k*, it is easy to get that

$$k\leq \left \{ \textstyle\begin{array}{l@{\quad}l} \frac{n-1}{2}& \mbox{when $n$ is odd},\\ \frac{n-2}{2}&\mbox{when $n$ is even}. \end{array}\displaystyle \right . $$

In total, we have $k \leq \lceil\frac{n-2.5}{2} \rceil+1$. □

### Remark 1

In Corollary 2, we obtained a relation of inequality between *n* and *k* under the assumption $h(0,A,k)<1$. Furthermore, Foucart [23, p. 49, Chapter 2] showed another relation of inequality between *m* and *k*. If every *k*-sparse vector $x\in\mathbb{R}^{n}$ can be recovered via $l_{0}$-minimization, then we get that $m\geq2k$; furthermore, it is easy to get that $k\leq \lceil\frac{m}{2} \rceil$ due to the integer values of *k*.

### Remark 2

Chen and Gu [22] showed some important properties of $h(p,A,k)$. It is shown that $h(p,A,k)$ is a continuous function in $p\in[0,1]$ when $k\leq \operatorname{spark}(A)-1$, where $\operatorname{spark}(A)$ is the smallest number of columns from *A* that are linearly dependent. Therefore, if $h(0,A,k)<1$ for some fixed *A* and *k*, then there exists a constant $p^{\ast}$ such that $h(p,A,k)<1$ for $p \in[0,p^{\ast})$, that is, every *k*-sparse vector can be recovered via both $l_{0}$-minimization and $l_{p}$-minimization for $p\in(0,p^{\ast})$, which is a corollary of the main theorem in [7].

### Theorem 2

([7])

*There exists a constant* $p(A,b)>0$ *such that when* $0< p< p(A,b)$, *every solution to* $l_{p}$-*minimization also solves* $l_{0}$-*minimization*.

Theorem 2 is the main theorem in [7]. Obviously, this theorem qualitatively proves the effectiveness of solving the original $l_{0}$-minimization problem via $l_{p}$-minimization. Moreover, the theorem becomes more practical if $p(A,b)$ is computable. At the end of this section, we need to point out a necessary and sufficient condition based on the $l_{p}$-null space property, and NSC can provide us the following lemma, which is similar to RIP.

### Lemma 1

*Given an underdetermined matrix* $A \in\mathbb{R}^{m \times n}$ *and an integer* *k*, *the inequality* $h(0,A,k)<1$ *holds if and only if there exist two constants* $0< u\leq w$ *with*

7 $$ 0< \lambda_{\min^{+}}\bigl(A^{T}A\bigr)\leq u^{2}\leq w^{2} \leq\lambda_{\max}\bigl(A^{T}A\bigr) $$

*such that*

8 $$ u^{2}\|x\|_{2}^{2} \leq\|Ax\|_{2}^{2} \leq w^{2}\|x\|_{2}^{2} $$

*for every* 2*k*-*sparse vector* $x\in\mathbb{R}^{n}$.

### Proof

*Necessity.* The proof is divided into two steps.

*Step 1*: Proof of the existence of *u*.

To prove this result, we just need to prove that the set

$$V=\bigl\{ u: \|Ax\|_{2}/\|x\|_{2} \geq u \mbox{ for any nonzero } x \mbox{ with } \|x\|_{0} \leq2k \bigr\} $$

has a nonzero infimum.

If we assume that inf $V=0$, then, for any $n \in N^{+}$, there exists a vector 2*k*-sparse vector $x_{n}$ with $\|x_{n}\|_{2}=1$ such that $\|Ax_{n}\| _{2} \leq n^{-1}$.

Furthermore, it is easy to get a convergent subsequence $\{x_{n_{i}}\}$ of the bounded sequence $\{x_{n}\}$, that is, $x_{n_{i}} \to x_{0}$, and it is obvious that $Ax_{0}=\mathbf{0}$ because the function $y(x)=Ax$ is continuous.

Let $J(x_{0})=\{i:(x_{0})_{i} \ne0\}$. There exists $N_{i}$ such that $(x_{n_{k}})_{i} \ne0$ when $k \geq N_{i}$ for any $i \in J(x_{0})$.

Let $N=\max _{i \in J(x_{0})}N_{i}$. For any $i \in J(x_{0})$, it is easy to get that $(x_{n_{k}})_{i} \ne0$ when $k \geq N$. When $k \geq N$, we get that $\|x_{n_{k}}\|_{0} \geq\|x_{0}\|_{0}$ and $\|x_{0}\|_{0} \leq2k$.

However, according to Corollary 2, it is easy to get that $\|x\|_{0} \geq 2k+1$ for any $x\in \operatorname{Ker}(A)\setminus\{\mathbf{0}\}$. We notice that $x_{0} \in \operatorname{Ker}(A)$, so the result $\|x_{0}\|_{0} \leq2k$ contradicts Corollary 2.

Therefore, there exists a constant $u>0$ such that $\|Ax\|_{2} \geq u\|x\| _{2}$ for any $x \in\mathbb{R}^{n}$ with $\|x\|_{0} \leq2k$.

*Step 2*: Proof of $u^{2} \geq\lambda_{\min^{+}}(A^{T}A)$.

According to the proof above, there exists a vector $\widetilde{x} \in \mathbb{R}^{n}$ with $\|\widetilde{x}\|_{0} \leq2k$ such that $\| A\widetilde{x}\|_{2}=u\| \widetilde{x}\|_{2}$.

Let $V=\operatorname{support} (\widetilde{x})$. It is easy to get that

9 $$ u^{2}x^{T}x \leq x^{T}A_{V}^{T}A_{V}x $$

for all $x \in\mathbb{R}^{|V|}$. Therefore, the smallest eigenvalue of $A_{V}^{T}A_{V}$ is $u^{2}$ since $A_{V}^{T}A_{V} \in R^{|V| \times|V|}$ is a symmetric matrix, and we can choose an eigenvector $z\in R^{|V|}$ of eigenvalue $u^{2}$.

If $u^{2}<\lambda_{\min^{+}}(A^{T}A)$, then consider the vector $x{'}\in \mathbb{R}^{n}$ with $x_{i}{'}=z_{i}$ when $i \in V$ and zero otherwise. Therefore, it is easy to get that $A^{T}Ax{'}=u^{2}x{'}$, which contradicts the definition of $\lambda_{\min^{+}}(A^{T}A)$.

Finally, notice that $A^{T}A$ is a semipositive definite matrix such that $\|Ax\|_{2}^{2}=x^{T}A^{T}Ax\leq\lambda_{\max}(A^{T}A)\|x\|_{2}^{2}$ for all $x\in \mathbb{R}^{n}$. So there exists a constant *w* such that $\|Ax\|_{2}^{2} \leq w^{2}\|x\|_{2}^{2} $ for all $\|x\|_{0} \leq2k$.

*Sufficiency.* Let a *k*-sparse vector $x^{\ast}$ be the unique solution of $l_{0}$-minimization. For any *k*-sparse vector $x_{1}$, we have that

10 $$ u^{2}\big\| x^{\ast}-x_{1}\big\| _{2}^{2} \leq\big\| A\bigl(x^{\ast}-x_{1}\bigr)\big\| _{2}^{2} \leq w^{2}\big\| x^{\ast}-x_{1}\big\| _{2}^{2}. $$

Therefore, we get that $x^{\ast}=x_{1}$ as long as $x_{1}$ is a solution of $Ax=b$, that is, every *k*-sparse vector can be recovered by $l_{0}$-minimization (1), and this is equivalent to $h(0,A,k)<1$ by Corollary 1. □

## Main contribution

In this section, we focus ourselves on the proposed problem. By introducing a new technique and utilizing preparations provided in Section 2, we will present an analytic expression of $p^{\ast}(A,b)$ such that every *k*-sparse vector *x* can be recovered via $l_{p}$-minimization with $0< p< p^{\ast}(A,b)$ as long as it can be recovered via $l_{0}$-minimization. To this end, we first begin with two lemmas.

### Lemma 2

*For any* $x\in\mathbb{R}^{n}$ *and* $0< p \leq1$, *we have that*

$$ \|x\|_{p} \leq\|x\|_{0}^{\frac{1}{p}-\frac{1}{2}}\|x \|_{2}. $$

### Proof

This result can be easily proved by Hölder’s inequality. □

### Lemma 3

*Given a matrix* $A \in\mathbb{R}^{m \times n}$, *if* $u\|x\|_{2} \leq\| Ax\|_{2} \leq w\|x\|_{2}$ *for all* $\|x\|_{0} \leq2k$, *then*

$$\big|\langle Ax_{1},Ax_{2}\rangle\big| \leq \frac{w^{2}-u^{2}}{2}\|x_{1}\| _{2}\|x_{2} \|_{2} $$

*for all* $x_{1}$ *and* $x_{2}$ *with* $\|x_{i}\|_{0} \leq k$ ($i=1,2$), *and* $\operatorname{support} (x_{1}) \cap \operatorname{support} (x_{2})= \varnothing$.

### Proof

By the assumption on the matrix *A*, it is easy to get that

11 $$\begin{aligned}[b] \frac{|\langle Ax_{1},Ax_{2} \rangle|}{\|x_{1}\|_{2}\|x_{2}\| _{2}}&= \biggl\vert \biggl\langle A \biggl( \frac{x_{1}}{\|x_{1}\| _{2}} \biggr), A \biggl(\frac{x_{2}}{\|x_{2}\|_{2}} \biggr) \biggr\rangle \biggr\vert \\ &=\frac{1}{4} \biggl\vert \biggl\Vert A \biggl( \frac{x_{1}}{\|x_{1}\| _{2}}+\frac{x_{2}}{\|x_{2}\|_{2}} \biggr) \biggr\Vert _{2}^{2}- \biggl\Vert A \biggl(\frac {x_{1}}{\|x_{1}\|_{2}}-\frac{x_{2}}{\|x_{2}\|_{2}} \biggr) \biggr\Vert _{2}^{2} \biggr\vert \\ &\leq\frac{1}{4} \biggl\vert w^{2} \biggl\Vert \frac{x_{1}}{\|x_{1}\|_{2}}+\frac {x_{2}}{\|x_{2}\|_{2}} \biggr\Vert _{2}^{2}-u^{2} \biggl\Vert \frac{x_{1}}{\|x_{1}\|_{2}}-\frac {x_{2}}{\|x_{2}\|_{2}} \biggr\Vert _{2}^{2} \biggr\vert .\end{aligned} $$

Since $\operatorname{support} (x_{1}) \cap \operatorname{support} (x_{2})= \varnothing$, we have that

12 $$ \biggl\Vert \frac{x_{1}}{\|x_{1}\|_{2}}+\frac{x_{2}}{\|x_{2}\|_{2}} \biggr\Vert _{2}^{2}= \biggl\Vert \frac{x_{1}}{\|x_{1}\|_{2}}-\frac{x_{2}}{\|x_{2}\|_{2}} \biggr\Vert _{2}^{2}=2, $$

from which we get that

13 $$ \big|\langle Ax_{1},Ax_{2} \rangle\big| \leq \frac{w^{2}-u^{2}}{2}\| x_{1}\|_{2}\|x_{2} \|_{2}. $$

 □

With the above lemmas in hand, we now can prove our main theorems.

### Theorem 3

*Given a matrix* $A \in\mathbb{R}^{m \times n}$ *with* $m \leq n$ *and* $0< p\leq1$, *if* $h(0,A,k)<1$, *then*

14 $$ h(p,A,k)< h^{\ast}(p,A,k), $$

*where*

15 $$ h^{*}(p,A,k)= \biggl(\frac{\sqrt{2} +1}{2} \biggr)^{p} \biggl(\frac{k}{k+1} \biggr) \biggl[\frac{(\lambda-1)(n-2-k)}{2k}+ \biggl(\lambda+\sqrt{\frac{1}{k+1}} \biggr)k^{- \frac{1}{2}} \biggr]^{p} $$

*with*

$$ \lambda= \frac{\lambda_{\max}(A^{T}A)}{\lambda _{\min^{+}}(A^{T}A)}. $$

### Proof

According to Theorem 1 and Corollary 2, it is easy to get that $\|x\|_{0} \geq2k+1$ for every $x \in \operatorname{Ker}(A)\setminus\{\mathbf{0}\}$ since $h(0,A,k)<1$. Furthermore, according to Lemma 1, we can find constants $\lambda_{\min^{+}}(A^{T}A)\leq u^{2}\leq w^{2}\leq\lambda_{\max}(A^{T}A)$ such that

16 $$ u\|\tilde{x}\|_{2} \leq\|A\tilde{x}\|_{2} \leq w\|\tilde{x} \|_{2} $$

for any $\tilde{x} \in\mathbb{R}^{n}$ with $\|\tilde{x}\|_{0} \leq2k$.

Now we consider a nonzero vector $x \in \operatorname{Ker}(A)\setminus\{\mathbf{0}\}$ and an arbitrary index set $\Omega_{0} \subset\{1,2,\ldots,n\}$ with $|\Omega_{0}|=k$. We partition the complement of $\Omega_{0}$ as $\Omega_{0}^{c}= \bigcup _{i=1}^{t} \Omega_{i}$, where

$$\begin{gathered} \Omega_{1}=\{\text{indices of the $k+1$ largest absolute-value components of $x-x_{\Omega_{0}}$}\}, \\ \Omega_{2}=\{\text{indices of the $k$ largest absolute-value components of $x-x_{\Omega_{0}}-x_{\Omega_{1}}$}\}, \\ \Omega_{3}=\{\text{indices of the $k$ largest absolute-value components of $x-x_{\Omega_{0}}-x_{\Omega_{1}}-x_{\Omega_{2}}$}\}, \\ \ldots \\ \Omega_{t}=\{\text{indices of the remaining components of $x$}\}.\end{gathered} $$

We know that $\|x\|_{0}\geq2k+1$, so both $\Omega_{1}$ and $\Omega_{0}$ are not empty, and there are only two cases:

(i) $\Omega_{0}$ and $\Omega_{i}$ ($i=2,\ldots, t-1$) all have *k* elements except, possibly, $\Omega_{t}$.
(ii) $\Omega_{0}$ has *k* elements, $\Omega_{1}$ has less than $k+1$ elements, and $\Omega_{i}$ ($i=2,\ldots, t-1$) are empty.

Furthermore, in both cases, the set $\Omega_{1}$ can be divided in two parts:

$$\begin{gathered} \Omega_{1}^{(1)}=\{\text{indices of the $k$ largest absolute-value components of $\Omega_{1}$} \}, \\ \Omega_{1}^{(2)}=\{\text{indices of the rest components of $\Omega_{1}$} \}.\end{gathered}$$

It is obvious that $\Omega_{1}=\Omega_{1}^{(1)} \cup\Omega_{1}^{(2)}$ and the set $\Omega_{1}^{(2)}$ is not empty since $\|x\|_{0}\geq2k+1$.

Since $u\|\tilde{x}\|_{2} \leq\|A\tilde{x}\|_{2} \leq w\|\tilde{x}\|_{2}$ for any $\|\tilde{x}\|_{0} \leq2k$, we have that

17 $$ \begin{aligned}[b] \|x_{\Omega_{0}}\|_{2}^{2}+ \|x_{\Omega_{1}}\|_{2}^{2}&=\| x_{\Omega_{0}} \|_{2}^{2}+\|x_{\Omega_{1}^{(1)}}\|_{2}^{2}+ \|x_{\Omega_{1}^{(2)}}\| _{2}^{2} \\ &=\|x_{\Omega_{0}}+x_{\Omega_{1}^{(1)}}\|_{2}^{2}+ \|x_{\Omega _{1}^{(2)}}\|_{2}^{2} \\ &\leq \frac{1}{u^{2}}\|A(x_{\Omega_{0}}+x_{\Omega _{1}^{(1)}}) \|_{2}^{2}+\big\| x_{\Omega_{1}^{(2)}}\big\| _{2}^{2}.\end{aligned} $$

Since $x=x_{\Omega_{0}}+x_{\Omega_{1}^{(1)}}+x_{\Omega _{1}^{(2)}}+x_{\Omega_{2}}+\cdots+x_{\Omega_{t}} \in \operatorname{Ker}(A)\setminus\{\mathbf{0}\}$, we have that

18 $$ \begin{aligned}[b]\big\| A(x_{\Omega_{0}}+x_{\Omega_{1}^{(1)}})\big\| _{2}^{2}={}& \bigl\langle A(-x_{\Omega_{0}}-x_{\Omega_{1}^{(1)}},A(x_{\Omega_{1}^{(2)}}+x_{\Omega _{2}}+ \cdots+x_{\Omega_{t}})\bigr\rangle \\ ={}&\bigl\langle A(-x_{\Omega_{0}}-x_{\Omega_{1}^{(1)}}),Ax_{\Omega _{1}^{(2)}} \bigr\rangle \\ & +\sum_{i=2}^{t}\bigl(\bigl\langle A(-x_{\Omega_{0}}),Ax_{\Omega _{i}}\bigr\rangle +\bigl\langle A(-x_{\Omega_{1}^{(1)}}),Ax_{\Omega_{i}}\bigr\rangle \bigr).\end{aligned} $$

According to Lemma 3, for any $i\in\{2,3,\ldots, t\}$, we get that

19 $$ \begin{gathered} \bigl\langle A(-x_{\Omega_{0}}),Ax_{\Omega_{i}} \bigr\rangle \leq\frac {w^{2}-u^{2}}{2}\|x_{\Omega_{0}}\|_{2} \|x_{\Omega_{i}}\|_{2}, \\ \bigl\langle A(-x_{\Omega_{1}^{(1)}}),Ax_{\Omega_{i}}\bigr\rangle \leq \frac {w^{2}-u^{2}}{2}\|x_{\Omega_{1}^{(1)}}\|_{2}\|x_{\Omega_{i}} \|_{2}. \end{gathered} $$

Substituting inequalities (19) into (18), we have

20 $$ \begin{aligned}[b]\big\| A(x_{\Omega_{0}}+x_{\Omega_{1}^{(1)}})\big\| _{2}^{2} \leq{}&\big\| A(-x_{\Omega_{0}}-x_{\Omega_{1}^{(1)}})\big\| _{2} \|Ax_{\Omega_{1}^{(2)}}\| \\ &+ \frac{w^{2}-u^{2}}{2} \Biggl(\sum_{i=2}^{t} \|x_{\Omega_{i}}\| _{2} \Biggr) \bigl(\|x_{\Omega_{0}}\|_{2}+ \|x_{\Omega_{1}^{(1)}}\|_{2}\bigr) \\ \leq{}& w^{2}\bigl(\|x_{\Omega_{0}}\|_{2}+\|x_{\Omega_{1}^{(1)}} \|_{2}\bigr)\|x_{\Omega _{1}^{(2)}}\|_{2} \\ &+\frac{w^{2}-u^{2}}{2} \Biggl(\sum_{i=2}^{t} \|x_{\Omega_{i}}\| _{2} \Biggr) \bigl(\|x_{\Omega_{0}}\|_{2}+ \|x_{\Omega_{1}^{(1)}}\|_{2}\bigr).\end{aligned} $$

By the definition of $x_{\Omega_{1}^{(1)}}$ and $x_{\Omega_{1}}$ it is easy to get that

21 $$ \|x_{\Omega_{1}^{(1)}}\|_{2} \leq\|x_{\Omega_{1}}\|_{2} $$

and

22 $$ \|x_{\Omega_{1}^{(2)}}\|_{2} \leq\sqrt{\frac{1}{k+1}}\| x_{\Omega_{1}}\|_{2}. $$

Therefore, we get that

23 $$ \|x_{\Omega_{1}^{(2)}}\|_{2}^{2} \leq \sqrt{\frac {1}{k+1}} \bigl(\|x_{\Omega_{0}}\|_{2}+\|x_{\Omega_{1}} \|_{2} \bigr)\| x_{\Omega_{1}^{(2)}}\|_{2}. $$

Substituting inequalities (20) and (23) into (17), we have that

24 $$ \begin{aligned}[b]\|x_{\Omega_{0}}\|_{2}^{2}+ \|x_{\Omega_{1}}\|_{2}^{2} \leq{} & \frac{1}{u^{2}}\big\| A(x_{\Omega_{0}}+x_{\Omega_{1}^{(1)}})\big\| _{2}^{2}+ \|x_{\Omega_{1}^{(2)}}\|_{2}^{2} \\ \leq{}& \bigl(\|x_{\Omega_{0}}\|_{2}+\|x_{\Omega_{1}} \|_{2}\bigr) \Biggl[ \frac{w^{2}-u^{2}}{2u^{2}} \sum _{i=2}^{t} \| x_{\Omega_{i}} \|_{2} \\ & + \biggl(\frac{w^{2}}{u^{2}}+\sqrt{\frac{1}{k+1}} \biggr) \|x_{\Omega _{1}^{(2)}}\|_{2} \Biggr].\end{aligned} $$

For any $2\leq i\leq t$ and any element *a* of $x_{\Omega_{i}}$, it is easy to get that $|a|^{p} \leq \frac{1}{k+1}\|x_{\Omega _{1}}\|_{p}^{p}$, so we have the inequalities:

25 $$ \|x_{\Omega_{i}}\|_{2}^{2} \leq k(k+1)^{-\frac{2}{p}}\|x_{\Omega_{1}}\|_{p}^{2} $$

and

26 $$ \|x_{\Omega_{1}^{(2)}}\|_{2}^{2} \leq(k+1)^{-\frac{2}{p}}\|x_{\Omega_{1}}\|_{p}^{2}. $$

Substituting inequalities (25) and (26) into (24), we derive that

27 $$ \begin{aligned}[b] \|x_{\Omega_{0}}\|_{2}^{2}+ \|x_{\Omega_{1}}\|_{2}^{2} \leq{}& \bigl(\|x_{\Omega_{0}}\| _{2}+\|x_{\Omega_{1}}\|_{2}\bigr) \biggl[\frac{w^{2}-u^{2}}{2u^{2}}k^{\frac {1}{2}}(k+1)^{- \frac{1}{p}}(t-1) \\ &+ \biggl(\frac{w^{2}}{u^{2}}+\sqrt{\frac{1}{k+1}} \biggr) (k+1)^{-\frac{1}{p}} \biggr]\|x_{\Omega_{1}}\|_{p}.\end{aligned} $$

Let $r=\frac{w^{2}}{u^{2}}$ and

$$ B= \biggl[\frac{w^{2}-u^{2}}{2u^{2}}k^{\frac {1}{2}}(k+1)^{ -\frac{1}{p}}(t-1)+ \biggl(\frac{w^{2}}{u^{2}}+\sqrt{\frac {1}{k+1}} \biggr) (k+1)^{-\frac{1}{p}} \biggr]\|x_{\Omega_{1}}\|_{p}. $$

Then we can rewrite inequality (27) as

28 $$ \|x_{\Omega_{0}}\|_{2}^{2}+\|x_{\Omega_{1}} \|_{2}^{2} \leq B\bigl(\|x_{\Omega_{0}}\| _{2}+ \|x_{\Omega_{1}}\|_{2}\bigr), $$

so that

$$\biggl(\|x_{\Omega_{0}}\|_{2}- \frac{B}{2} \biggr)^{2}+ \biggl(\|x_{\Omega_{1}}\|_{2}- \frac{B}{2} \biggr)^{2}\leq \frac{B^{2}}{2}. $$

Therefore, we get that $\|x_{\Omega_{0}}\|_{2} \leq \frac {\sqrt{2}+1}{2}B$.

According to Lemma 2, we have that

29 $$ \|x_{\Omega_{0}}\|_{p} \leq k^{\frac{1}{p}-\frac{1}{2}}\| x_{\Omega_{0}}\|_{2} \leq k^{\frac{1}{p}-\frac{1}{2}} \frac{\sqrt{2}+1}{2}B. $$

Substituting the expression of *B* into inequality (29), we obtain that

30 $$ \begin{aligned}[b]\|x_{\Omega_{0}}\|_{p} \leq{}& k^{\frac{1}{p}-\frac {1}{2}} \biggl(\frac{\sqrt{2}+1}{2} \biggr) \\ &\times \biggl[\frac{r-1}{2}k^{\frac{1}{2}}(k+1)^{-\frac {1}{p}}(t-1)+ \biggl(r+\sqrt{\frac{1}{k+1}} \biggr) (k+1)^{-\frac {1}{p}} \biggr] \|x_{\Omega_{1}}\|_{p} \\ ={} & \biggl({\frac{k}{k+1}} \biggr)^{\frac{1}{p}} \biggl( \frac{\sqrt {2}+1}{2} \biggr) \biggl[\frac{r-1}{2}(t-1)+ \biggl(r+\sqrt{ \frac{1}{k+1}} \biggr)k^{-\frac{1}{2}} \biggr]\| x_{\Omega_{1}} \|_{p}.\end{aligned} $$

We notice that the sets $\Omega_{0}$ and $\Omega_{i}$ ($i=2,\ldots, t-1$) all have *k* elements and the set $\Omega_{1}$ has $k+1$ elements such that $tk+2 \leq n \leq(t+1)k+1$, so we get that $t \leq\frac{n-2}{k}$.

According to Lemma 1, we have that $r=\frac{w^{2}}{u^{2}} \leq\lambda =\frac{\lambda_{\max}(A^{T}A)}{\lambda_{\min^{+}}(A^{T}A)}$. Substituting the inequalities into (30), we obtain

$$ \|x_{\Omega_{0}}\|_{p}\leq \frac{\sqrt{2} +1}{2} \biggl(\frac{k}{k+1} \biggr)^{\frac{1}{p}} \biggl[\frac{(\lambda -1)(n-2-k)}{2k}+ \biggl(\lambda+\sqrt{\frac{1}{k+1}} \biggr)k^{- \frac{1}{2}} \biggr] \|x_{\Omega_{1}}\|_{p}. $$

It is obvious that $\|x_{\Omega_{1}}\|_{p}\leq\|x_{\Omega_{0}^{c}}\|_{p}$, and therefore we get that

$$\|x_{\Omega_{0}}\|_{p}^{p} \leq h^{\ast}(p,A,k) \|x_{\Omega_{0}^{c}}\|_{p}^{p}, $$

where $h^{\ast}(p,A,k)$ is given in (15).

According to the definition of $h(p,A,k)$, we can get that $h(p,A,k)\leq h^{\ast}(p,A,k)$. □

Theorem 3 presents a result that is very similar to the result in Theorem 1. However, it is worth pointing out that the constant $h^{\ast }(p,A,k)$ plays a central role in Theorem 3. In fact, we can treat $h^{\ast}(p,A,k)$ as an estimate of $h(p,A,k)$, where the former is calculable, and since the latter is NP-hard, $h^{\ast}(p,A,k)$ may be considered as an improvement of $h(p,A,k)$. According to Theorem 1, if we take *k* as the $l_{0}$-norm of the unique solution of $l_{0}$-minimization, then we can get the main contribution as soon as the inequality $h^{\ast}(p,A,k)^{\frac{1}{p}}<1$ is satisfied.

### Theorem 4

*Let* $A \in\mathbb{R}^{m \times n}$ *be an underdetermined matrix of full rank*, *and denote* $\Omega^{*}=|\operatorname{support} (A^{T}(AA^{T})^{-1}b)|$. *If every* *k*-*sparse vector* *x* *can be recovered via* $l_{0}$-*minimization*, *then* *x* *also can be recovered via* $l_{p}$-*minimization with* $p\in (0,p^{*}(A,b))$, *where*

31 $$ p^{*}(A,b)=\max \biggl\{ h \bigl(\Omega^{*} \bigr),h \biggl( \biggl\lceil \frac{n-2.5}{2} \biggr\rceil +1 \biggr),h \biggl( \biggl\lceil \frac{m}{2} \biggr\rceil \biggr) \biggr\} $$

*with*

32 $$ h(x)=\frac{\ln(x+1)-\ln x}{\ln [ ( \frac{\sqrt{2}+1}{2} ) ( \frac {(\lambda-1)(n-3)}{2}+\lambda+ \sqrt{\frac{1}{2}} ) ]} $$

*and*

$$ \lambda= \frac{\lambda_{\max}(A^{T}A)}{\lambda _{\min^{+}}(A^{T}A)}. $$

### Proof

Recalling (15), we can get the equivalence between $l_{0}$-minimization and $l_{p}$-minimization as long as $h^{\ast }(p,A,k)^{\frac{1}{p}}<1$. However, *k* cannot be calculated directly, and we need to estimate *k* and change the inequality $h^{\ast }(p,A,k)^{\frac{1}{p}}<1$ into a computable one through inequality technique.

Due to the integer values of $\|x\|_{0}$, we have that

$$\biggl(\lambda+\sqrt{ \frac{1}{k+1}} \biggr)k^{-\frac {1}{2}} \leq\lambda+\sqrt{ \frac{1}{2}}. $$

Notice that $\lambda>1$, so we have

$$\frac{(\lambda-1)(n-2-k)}{2k}\leq \frac {(\lambda-1)(n-3)}{2}. $$

Furthermore, according to Corollary 2, we get that $2k+1\leq n$, and it is easy to get that $(\lambda-1)(n-2-k)\geq0$, so that

$$h^{\ast}(p,A,k)^{\frac{1}{p}} \leq\frac{\sqrt {2}+1}{2} \biggl(\frac{k}{k+1} \biggr)^{\frac{1}{p}} \biggl[\frac {(\lambda-1)(n-3)}{2}+ \lambda+\sqrt{\frac{1}{2}} \biggr]. $$

Furthermore, it is obvious that $\frac{(\lambda-1)(n-3)}{2}+\lambda+ \sqrt{\frac{1}{2}}>0$. Therefore, for $x\in(0,+\infty)$ and $p \in (0,1)$, it is easy to prove that the function

$$f(x,p)=\frac{\sqrt{2}+1}{2} \biggl(\frac{x}{x+1} \biggr)^{\frac{1}{p}} \biggl[\frac{(\lambda-1)(n-3)}{2}+\lambda+\sqrt { \frac{1}{2}} \biggr] $$

increases in *x* when *p* is fixed and also increases in *p* when *x* is fixed.

According to Corollary 2 and Remark 2, we have that $k \leq \lceil\frac{n-2.5}{2} \rceil+1$, $k\leq \lceil\frac {m}{2} \rceil$, and $k \leq|\Omega^{\ast}|$, where $\Omega ^{\ast}=|\operatorname{support} (A^{T}(AA^{T})^{-1}b)|$, because it is obvious that $x=A^{T}(AA^{T})^{-1}b$ is a solution of the underdetermined system $Ax=b$.

Therefore, we get that

33 $$ f(k,p) \leq \min \biggl\{ f \biggl( \biggl\lceil \frac {n-2.5}{2} \biggr\rceil +1 ,p \biggr), f\bigl(\Omega^{\ast} ,p\bigr),f \biggl( \biggl\lceil \frac{m}{2} \biggr\rceil ,p \biggr) \biggr\} . $$

It is obvious that $f(k,p)<1$ as long as one of three inequalities $f ( \lceil\frac{n-2.5}{2} \rceil+1 ,p )<1$, $f(\Omega^{\ast} ,p)<1$, and $f ( \lceil\frac{m}{2} \rceil,p )<1$ is satisfied.

Furthermore, the inequality $f(x,p)<1$ when *x* fixed is very easily solved, and the range of such *p* is

$$p< h(x)= \frac{\ln(x+1)-\ln x}{\ln [ ( \frac{\sqrt{2}+1}{2} ) ( \frac {(\lambda-1)(n-3)}{2}+\lambda+ \sqrt{\frac{1}{2}} ) ]}. $$

Hence, for any $0< p< p^{\ast}=\max \{h(\Omega^{*}),h ( \lceil\frac{n-2.5}{2} \rceil+1 ),h ( \lceil \frac{m}{2} \rceil ) \}$, we have that $h^{\ast }(p,A,k)^{\frac{1}{p}} \leq f(k,p)<1$. Therefore, according to Theorem 1, every *k*-sparse vector $x\in\mathbb{R}^{n}$ can be recovered via both $l_{0}$-minimization and $l_{p}$-minimization. □

Combining Theorems 3 and 4, we have reached the major goals of this paper. The most important result in these two theorems is the analytic expression of $p^{\ast}(A,b)$, with which the specific range of *p* can be easily calculated.

Next, we present two examples to demonstrate the validation of Theorem 4. We consider two matrixes of different dimensions of their null spaces and get the unique solution to $l_{p}$-minimization to verify whether it is the unique solution to $l_{0}$-minimization.

### Example 1

We consider an underdetermined system $Ax=b$, where

$$A=\left ( \textstyle\begin{array}{c@{\quad}c@{\quad}c@{\quad}c} 1 & -1.5 & -0.7 & 0 \\ 0 & -2 & 0.5 & 1 \\ 1 & 0.5 & -1 & 1 \end{array}\displaystyle \right )$$

and

$$b=\left ( \textstyle\begin{array}{c} 0.5\\ 0\\ 0.5 \end{array}\displaystyle \right ).$$

It is obvious that the sparsest solution is $x^{\ast}=[0.5, 0, 0, 0]^{T}$ and $\operatorname{Ker}(A)$ is spanned by $[-10, -2, -10, 1]^{T}$, so the solutions of the equation $Ax=b$ can be expressed in the form

$$x=[0.5, 0, 0, 0]^{T}+t[-10, -2, -10, 1]^{T}, \quad \mbox{where } t\in \mathbb{R}. $$

Therefore, the *p*-norm of *x* can be expressed as

$$\|x\|_{p}^{p}=|0.5-10t|^{p}+|2t|^{p}+|10t|^{p}+|t|^{p}. $$

Furthermore, it is easy to get that $\lambda_{\max}(A^{T}A)=7.2583$, $\lambda_{\min}(A^{T}A)=1.1926$, and $\lambda=\frac{\lambda_{\max }(A^{T}A)}{\lambda_{\min}(A^{T}A)}=6.0856$.

We can get that

$$A^{T}\bigl(AA^{T}\bigr)^{-1}b=[0.2561, -0.0488, -0.2439, 0.0244]^{T}, $$

and hence $h(\Omega^{\ast})=0.0921$ and $h ( \lceil\frac {n-2.5}{2} \rceil+1 )= h ( \lceil\frac{m}{2} \rceil )=0.2862$, so $p^{\ast}(A,b)=0.2862$.

**Figure 1:**
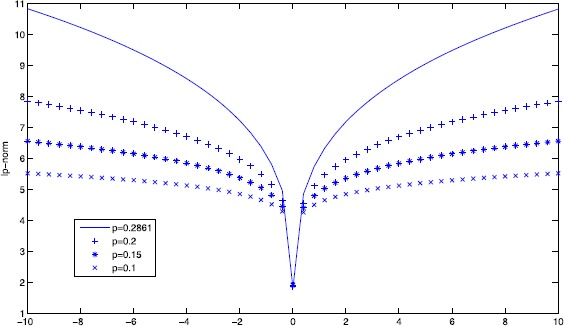
**The** ***p*** **-norm of the solutions of the different cases in Example** **1** **.**

As shown in the Figure 1, we can get the solution of $l_{p}$-minimization in different cases where $p=0.2861,0.2,0.15\text{, and }0.1$. It is obvious that $l_{0.2861}$-minimization, $l_{0.2}$-minimization, $l_{0.15}$-minimization, and $l_{0.1}$-minimization all reach their minimums at $t=0$, which corresponds to the sparsest solution $x^{\ast }=[0.5, 0, 0, 0]^{T}$.

### Example 2

We consider a more complex situation with $Ax=b$, where

$$A=\left ( \textstyle\begin{array}{c@{\quad}c@{\quad}c@{\quad}c@{\quad}c} 1 & 0 & 3.5 & -3 & -2.7 \\ 0 & 2 & 0 & -1.5 & 4.5 \\ 2 & 2 & -4 & -0.5 & 1.5 \end{array}\displaystyle \right ) $$

and

$$b=\left ( \textstyle\begin{array}{c} 0.5\\ 0\\ 1 \end{array}\displaystyle \right ). $$

It is easy to get the sparsest solution $x^{\ast}=[0.5, 0, 0, 0, 0]^{T}$, and the solutions of the underdetermined system $Ax=b$ can be expressed in the parameterized form

$$x=[0.5, 0, 0, 0, 0]^{T}+s \biggl[\frac{213}{110}, - \frac {9}{4}, \frac{12}{55}, 0, 1 \biggr]^{T}+t \biggl[ \frac{17}{22}, \frac{3}{4}, \frac{7}{11}, 1, 0 \biggr]^{T}, \quad \mbox{where } s,t\in\mathbb{R}.$$

Therefore

$$\|x\|_{p}^{p}= \biggl\vert 0.5+\frac{213}{110}s+ \frac{17}{22}t \biggr\vert ^{p}+ \biggl\vert - \frac{9}{4}s+\frac{3}{4}t \biggr\vert ^{p}+ \biggl\vert \frac{12}{55}s+\frac {7}{11}t \biggr\vert ^{p}+|s|^{p}+|t|^{p}. $$

Furthermore, we can get $\lambda=\frac{\lambda_{\max }(A^{T}A)}{\lambda_{\min}(A^{T}A)}=4.1792$. It is easy to get that

$$A^{T}\bigl(AA^{T}\bigr)^{-1}b=[0.1903, 0.1083, -0.1188, -0.1527, -0.0990]^{T}. $$

Hence $h(\Omega^{\ast})=0.0801$, $h ( \lceil\frac {n-2.5}{2} \rceil+1 )=0.1782$, and $h ( \lceil \frac{m}{2} \rceil )=0.3046$, so we take $p^{\ast}(A,b)=0.3046$.

**Figure 2:**
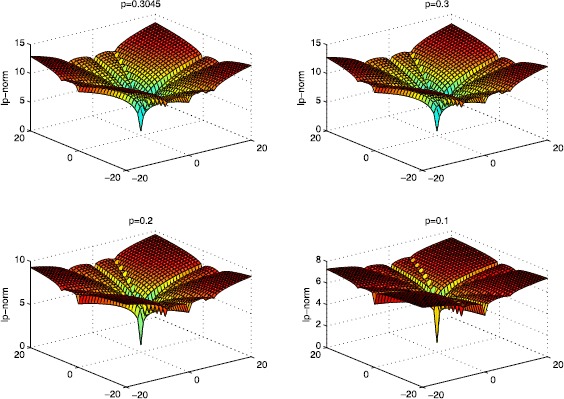
**The** ***p*** **-norm of the solutions of the different cases in Example** **2** **.**

**Figure 3:**
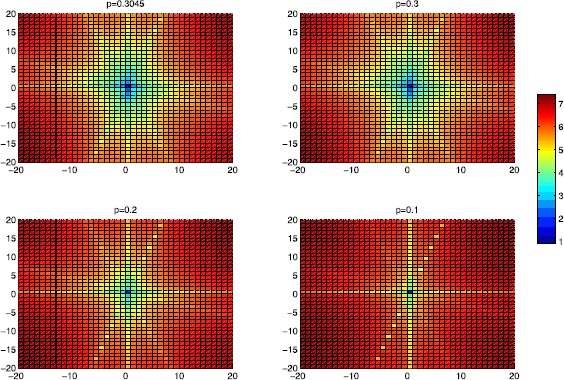
**On** ***s*** **-** ***t*** **plane for the** ***p*** **-norm of the solutions to the different cases in Example** **2** **.**

From Figure 2 we can also find the solutions in different cases where $p=0.3045, 0.3, 0.2\text{, and }0.1$. It is obvious that the minimum is reached at $s=t=0$, which corresponds to the sparsest solution $x^{\ast }=[0.5, 0, 0, 0, 0]^{T}$. The result can be seen more clearly in Figure 3.

## Conclusions

In this paper, we have studied the equivalence between $l_{0}$-minimization and $l_{p}$-minimization. By using the $l_{p}$-null space property and a sufficient and necessary condition to recover a sparse vector via these two models, we present an analytic expression of $p^{\ast}(A,b)$ such that $l_{p}$-minimization is equivalent to $l_{0}$-minimization. Although it is NP-hard to find the global optimal solution of $l_{p}$-minimization, a local minimizer can be done in polynomial time [24]. Chen [22] proved that $h(p,A,k)<1$ is a necessary and sufficient condition for the global optimality of $l_{p}$-minimization. Therefore, it is confident that we can find the sparse solution with $l_{p}$-minimization with $0< p< p^{\ast}(A,b)$ as long as we start with a good initialization.

However, in this paper, we only consider the situation where $l_{0}$-minimization has an unique solution. The uniqueness assumption is vital for us to prove the main results. However, from Lemma 1 we see that the uniqueness assumption is equivalent to a certain double-inequality condition, which looks like RIP. The evident difference between them is in that the former possesses the homogeneity rather than the latter. This implies that, unlike RIP, the uniqueness assumption is not in essential conflict with equivalence of all linear systems $\lambda Ax=\lambda x$, $\lambda\in\mathbb{R}$. Therefore, we think that the uniqueness assumption and, equivalently, the resulting double-inequality condition in Lemma 1 can replace the RIP in many cases.

## References

[1] Olshausen B, Field DJ (1996). Emergence of simple-cell receptive field properties by learning a sparse code for natural images. Nature.

[2] Candes EJ, Recht B (2009). Exact matrix completion via convex optimization. Found. Comput. Math..

[3] Malioutov D, Cetin M, Willsky AS (2005). A sparse signal reconstruction perspective for source localization with sensor arrays. IEEE Trans. Signal Process..

[4] Wright J, Ganesh A, Zhou Z (2009). Robust face recognition via sparse representation. IEEE Trans. Pattern Anal. Mach. Intell..

[5] Candes EJ, Tao T (2005). Decoding by linear programming. IEEE Trans. Inf. Theory.

[6] Chen L, Gu Y (2015). Local and global optimality of $l_{p}$ minimization for sparse recovery. IEEE International Conference on Acoustics, Speech and Signal Processing.

[7] Peng J, Yue S, Li H (2015). $\mathit{NP}/\mathit{CMP}$, equivalence: a phenomenon hidden among sparsity models, $l_{0}$-minimization and $l_{p}$-minimization for information processing. IEEE Trans. Inf. Theory.

[8] Natarajan BK (2006). Sparse approximate solutions to linear systems. SIAM J. Comput..

[9] Candes EJ (2008). The restricted isometry property and its implications for compressed sensing. C. R. Math..

[10] Candes EJ, Tao T (2006). Near-optimal signal recovery from random projections: universal encoding strategies?. IEEE Trans. Inf. Theory.

[11] Foucart S, Lai MJ (2009). Sparsest solutions of underdetermined linear systems via $l_{q}$-minimization for $0 < q\leq1$. Appl. Comput. Harmon. Anal..

[12] Donoho DL, Tanner J (2005). Sparse nonnegative solution of underdetermined linear equations by linear programming. Proc. Natl. Acad. Sci. USA.

[13] Tropp JA (2004). Greed is good: algorithmic results for sparse approximation. IEEE Trans. Inf. Theory.

[14] Petukhov A (2008). Fast implementation of orthogonal greedy algorithm for tight wavelet frames. Signal Process..

[15] Gao Y, Ma M (2017). A new bound on the block restricted isometry constant in compressed sensing. J. Inequal. Appl..

[16] Gribonval R, Nielsen M (2004). Sparse representations in unions of bases. IEEE Trans. Inf. Theory.

[17] Cai TT, Zhang A (2013). Sparse representation of a polytope and recovery of sparse signals and low-rank matrices. IEEE Trans. Inf. Theory.

[18] Chartrand R (2007). Exact reconstruction of sparse signals via nonconvex minimization. IEEE Signal Process. Lett..

[19] Sun Q (2010). Recovery of sparsest signals via $l_{p}$-minimization. Appl. Comput. Harmon. Anal..

[20] Daubechies I, Devore R, Fornasier M (2008). Iteratively reweighted least squares minimization for sparse recovery. Commun. Pure Appl. Math..

[21] Gao Y, Peng J, Yue S, Zhao Y (2015). On the null space property of $l_{q}$-minimization for $0 < q\leq1$ in compressed sensing. J. Funct. Spaces.

[22] Chen L, Gu Y (2015). On the null space constant for $l _{p}$-minimization. IEEE Signal Process. Lett..

[23] Foucart S, Rauhut H (2013). A Mathematical Introduction to Compressive Sensing.

[24] Ge D, Jiang X, Ye Y (2011). A note on the complexity of $l_{p}$-minimization. Math. Program..

